# Incorporating adaptive genomic variation into predictive models for invasion risk assessment

**DOI:** 10.1016/j.ese.2023.100299

**Published:** 2023-07-11

**Authors:** Yiyong Chen, Yangchun Gao, Xuena Huang, Shiguo Li, Zhixin Zhang, Aibin Zhan

**Affiliations:** a Research Center for Eco-Environmental Sciences, Chinese Academy of Sciences, Beijing, 100085, China; b Guangdong Key Laboratory of Animal Conservation and Resource Utilization, Guangdong Public Laboratory of Wild Animal Conservation and Utilization, Institute of Zoology, Guangdong Academy of Science, Guangzhou, 510260, China; c University of Chinese Academy of Sciences, Chinese Academy of Sciences, Beijing, 100049, China; d CAS Key Laboratory of Tropical Marine Bio-resources and Ecology, South China Sea Institute of Oceanology, Chinese Academy of Sciences, Guangzhou, 510275, China; e Global Ocean and Climate Research Center, South China Sea Institute of Oceanology, Chinese Academy of Sciences, Guangzhou, 510275, China

**Keywords:** Invasion risk, Climate change, Adaptive genomic variation, Genomic offset, Habitat suitability

## Abstract

Global climate change is expected to accelerate biological invasions, necessitating accurate risk forecasting and management strategies. However, current invasion risk assessments often overlook adaptive genomic variation, which plays a significant role in the persistence and expansion of invasive populations. Here we used *Molgula manhattensis*, a highly invasive ascidian, as a model to assess its invasion risks along Chinese coasts under climate change. Through population genomics analyses, we identified two genetic clusters, the north and south clusters, based on geographic distributions. To predict invasion risks, we employed the gradient forest and species distribution models to calculate genomic offset and species habitat suitability, respectively. These approaches yielded distinct predictions: the gradient forest model suggested a greater genomic offset to future climatic conditions for the north cluster (i.e., lower invasion risks), while the species distribution model indicated higher future habitat suitability for the same cluster (i.e, higher invasion risks). By integrating these models, we found that the south cluster exhibited minor genome-niche disruptions in the future, indicating higher invasion risks. Our study highlights the complementary roles of genomic offset and habitat suitability in assessing invasion risks under climate change. Moreover, incorporating adaptive genomic variation into predictive models can significantly enhance future invasion risk predictions and enable effective management strategies for biological invasions in the future.

## Highlights

• We found distinct risks using species distribution vs. gradient forest models.
• Habitat suitability change and genomic offset are functionally complementary.
• The genomic-niche index provides more comprehensive predictions of invasion risks.
• We recommend the incorporation of adaptative genomic variation into risk assessment.

## Graphical abstract

**Figure:**
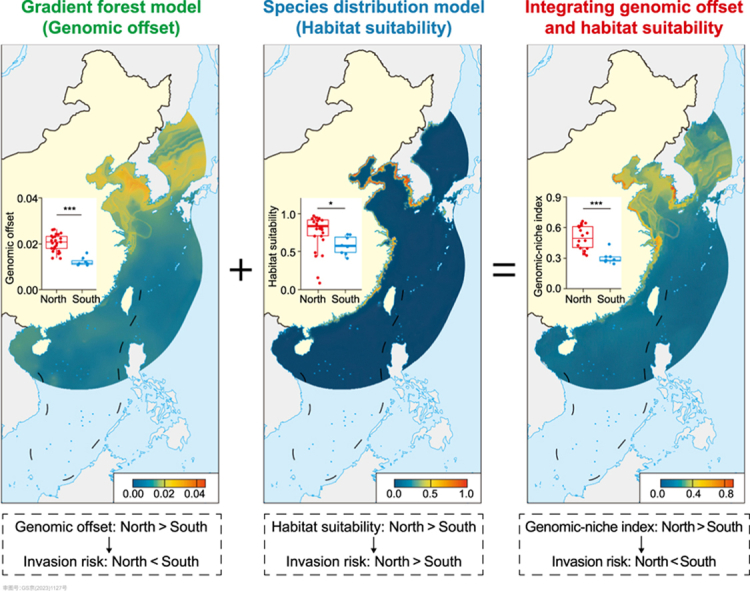

## 1 Introduction

Biological invasion and climate change are two major environmental threats to global biodiversity, and both have caused severely negative ecological, economic, and social consequences [1,2]. Importantly, the extent and intensity of biological invasions are expected to exacerbate under global climate change, particularly in marine and coastal ecosystems [3,4]. Marine ecosystems are extremely susceptible to biological invasions, as frequent human activities, such as shipping coupled with climate change, enable marine species to overcome geographical barriers and facilitate their wide geographical spread [5]. Therefore, it is urgent to assess invasion risks and design effective management at the early stages of biological invasions in marine and coastal ecosystems.

Species distribution models (SDMs; abbreviations were listed in Table S5) can predict species habitat suitability by linking species distribution data to environmental predictors [6]. SDMs represent an effective tool to assess geographical patterns of invasion risks under current and future climatic scenarios [[6], [7], [8]]. Despite their popularity, SDMs are based on several critical assumptions, including the niche conservatism hypothesis, which assumes that all populations within a species are spatial homogeneity and respond uniformly to climate change [6]. Recent empirical studies have challenged this hypothesis, revealing evidence of niche shifts during biological invasions, particularly in marine invasive species [9]. Relative studies have detected remarkable divergence among populations within a single invasive species, suggesting that rapid adaptation to diverse local environments plays a crucial role during range expansions [[10], [11], [12]]. Therefore, it is critical to consider intraspecific variation when assessing invasion risks using SDMs.

The incorporation of genomic information into SDMs provides a possible solution for this critical inherent limitation [13]. A commonly used approach is to incorporate spatial genetic structure into SDMs, which can be achieved by identifying genetic clusters and developing distribution models for individual clusters [14,15]. Despite its usefulness, this approach ignores the genomic information in relation to rapid evolution during invasions [13]. More specifically, as the frequency of alleles varies across populations, the genotype-environment matching pattern indicates the signature of genomic adaptation to local environments or climate change [16]. To overcome these limitations, researchers have attempted to map species genomic variation at landscape scales *via* modeling approaches such as the gradient forest model [17]. This new approach makes it possible to evaluate the disruption of gene-environment relationships under climate change scenarios (termed as “genetic offset”) [17]. Thus far, genetic offset has been widely promoted by genomic techniques (known as “genomic offset”) and applied in a variety of taxa, such as forest tree species [18] and terrestrial animals [19,20]. However, its implementation in marine species remains limited, with only a few studies exploring its potential in those taxa (e.g., Ref. [21]). Besides, integrating genomic offset into the invasion risk assessment of alien species under climate change is still in its infancy.

Invasive ascidians provide good models for studying invasion success in marine and coastal ecosystems [22]. *Molgula manhattensis* is native to the east coast of North America and has widely invaded global coastal ecosystems [23,24]. Many biological features of this invasive ascidian, such as high fecundity, strong competitive capacity, and a high potential for rapid evolution, are believed to enable its rapid adaptation to varied local environmental conditions during invasions [24]. After an initial introduction into Chinese coasts, the invasion fronts have been pushed forward onto dramatically different local environments from the north, such as the Bohai Sea, and the south, such as the Southern Yellow Sea [25]. Thus far, both field observations and SDM assessments have shown the ongoing and future spread of this invader at regional and global scales [26]. However, the impact of genomic variation, particularly those environmental adaptation-related, on the future invasions of *M. manhattensis* remains unclear, especially along Chinese coasts that are experiencing rapid and profound climate change.

In this study, we aim to assess the invasion risks of *M. manhattensis* under future climate scenarios by incorporating genomic variation and species habitat suitability. To achieve this, we implemented a three-step approach. Firstly, we identified environment-associated genomic variation and utilized gradient forest models to calculate the genomic offset, enabling the prediction of population vulnerability to future climate conditions. Secondly, we developed SDMs to predict the habitat suitability of this species along Chinese coasts under future climate change. Finally, we comprehensively integrated the genomic offset and habitat suitability to estimate future invasion risks.

## 2 Materials and methods

### 2.1 Environment-associated genomic variation

**Fig. 1:**
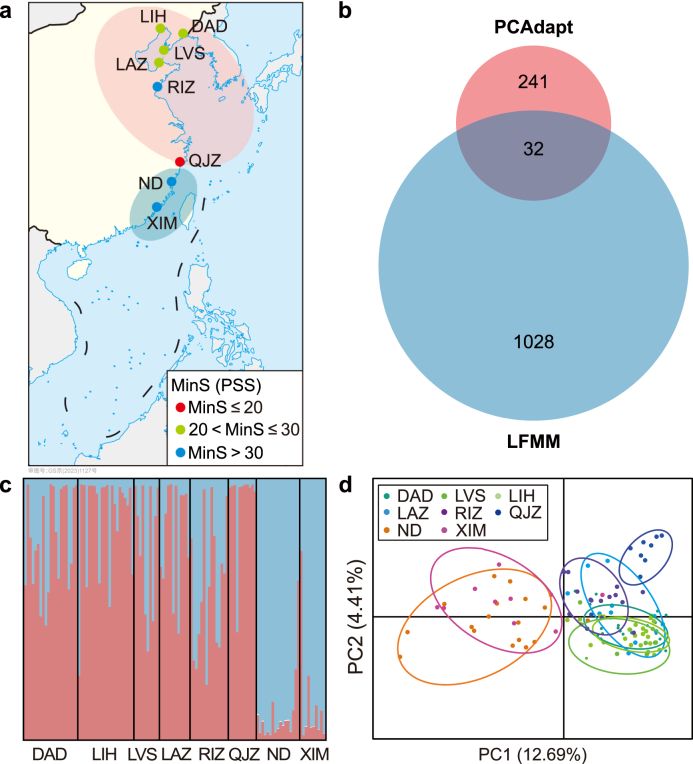
Two adaptive genetic clusters identified in the highly invasive *Molgula manhattensis* along Chinese coasts. **a**, Eight sampling sites along Chinese coasts. MinS indicates the lowest monthly average salinity. **b**, Adaptive single nucleotide polymorphisms (SNPs) identified by PCAdapt and LFMM. **c**, Population genetic structure based on adaptive single nucleotide polymorphisms (SNPs) using STRUCTURE. **d**, Population genetic structure based on adaptive single nucleotide polymorphisms (SNPs) using PCA.

We collected a total of 118 *M. manhattensis* individuals from eight sites along Chinese coasts (Fig. 1a). Genome-wide single nucleotide polymorphisms (SNPs) were obtained with the reduced-representation genome sequencing (2b-RAD), and detailed information, including sample collection, genomic DNA extraction, and genomic data generation and processing, was provided in our previous project [24].

We chose outlier detection methods, including correction steps for population structure, i.e., one population differentiation-based method (PCAdapt) and one environmental association-based method (LFMM, latent factor mixed-effect model). This strategy has been widely used in related studies to reduce the confounding effects of demographic history when investigating the signature of environmental adaptation [10,16,20]. Firstly, we employed the “PCAdapt” package in R to identify potential outlier SNPs indicative of selection [27]. We employed a K value of 5 in PCAdapt to accommodate population genetic structure, consistent with our previous findings based on STRUCTURE and DAPC analyses [24]. SNPs with a false discovery rate (FDR) lower than 0.05 were considered candidate outliers (*q*-value < 0.05). Secondly, for the environmental association approach, we adopted LFMM using the R package “LEA” [28]. LFMM implemented a hierarchical Bayesian mixed model to correct for unobserved latent factors and has been widely used to identify SNPs significantly associated with environmental variables [10,24,29]. The number of latent factors was also set to five, as inferred from the previous population genetic structure [24]. We retrieved various marine environmental layers from the Bio-ORACLE v2.0 database (https://www.bio-oracle.org) [30], which is extensively used in marine macroecological studies [31]. We focused on sea surface temperature and salinity, which are well known to exert considerable influence on the ecological and physiological performance of marine species, including ascidians [[22], [23], [24],^32^]. We extracted 12 environmental variables under present scenarios, including annual mean, maximum, minimum, range, and the average of the maximum and minimum values per year of sea surface temperature and salinity. While the Bio-ORACLE database contains predictors related to current velocity, we excluded these variables due to the limited dispersal ability of ascidians [22,24] and recent evidence suggesting minor effects of current velocity on ascidians distribution [26]. As LFMM does not address collinearity among environmental variables, we first conducted principal component analysis (PCA) and retained higher-order PCs (i.e., the sum of PCs > 90%), followed by the developers’ guidance [28]. The *lfmm* function in R package “LEA” was conducted with 10,000 iterations for five replicates, with 5,000 steps for burn-in. SNPs with median *z*-scores exceeding the absolute value of two [abs(*z*-score) > 2] and *q*-values lower than 0.05 adjusted for multiple testing were considered significantly environment-associated. SNPs identified by at least one of the two approaches (PCAdapt and LFMM) were considered putatively adaptive SNPs [10,24].

To characterize genetic composition among populations based on the putatively adaptive SNPs we used two population assignment analyses: STRUCTURE and PCA. The STRUCTURE analysis was conducted with the R package “LEA”, which can estimate individual admixture coefficients and evaluate the number of ancestral populations using the sparse non-negative matrix factorization (sNMF) [28]. The sNMF analysis was repeated ten times for each *K* value between 1 and 8, and the number of sNMF clusters with the lowest cross-entropy value was selected as the optimal value. PCA was performed with the R package “SNPRelate” [33].

We used redundancy analysis (RDA) to test the influence of environmental, geographical, and spatial variables on population genomic variation. Apart from the above 12 temperature and salinity-related predictors, we incorporated two geographical predictors, namely water depth and distance to shore, downloaded from the Global Marine Environment Datasets (http://gmed.auckland.ac.nz). Ascidians are known to exhibit a predominant distribution in near-shore shallow waters [22,23], and these two geographical predictors can affect their distributions [26]. Previous empirical studies on ascidians have revealed that extreme environmental conditions, such as low salinity and high temperature, were detrimental to their survival, growth, and geographical distributions [34,35]. To address collinearity among variables, we calculated the pairwise Pearson’s correlation coefficient (*r*) and retained one predictor among highly correlated variables (|*r*| > 0.70) (Fig. S1) [36]. Additionally, we incorporated principal coordinates of neighborhood matrices (PCNMs) as predictors for spatial structuring and unmeasured environmental variables. PCNMs were calculated from the geographic coordinates of each sampling site using the *pcnm* function in the R package “vegan”, and the first two uncorrelated spatial eigenfunctions (PCNM1 and PCNM2) were retained [24]. Based on biological importance and collinearity analysis results, our initial RDA model included six predictors: minimum sea surface salinity, maximum sea surface temperature, the annual range of sea surface temperature, water depth, distance to shore, and PCNM2 (excluding PCNM1 due to high collinearity with other predictors above). The allele frequency of putatively adaptive SNPs was detrended using the *decostand* function with the *hellinger* method implemented in the R package “vegan”. Significant explanatory variables were selected by using the *forward.sel* function in R package “packfor”, and these variables were used to develop a parsimonious RDA model with the highest adjusted coefficient of determination (*R*_adj_^2^).

### 2.2 Gradient forest model to predict genomic offset

The gradient forest, a regression tree-based machine learning algorithm, was employed to test nonlinear associations among spatial, environmental, and allelic variables [17]. Similarly to RDA, the gradient forest model included six predictors. To assess the robustness of gradient forest models, we compared two different sets of SNPs: (i) the complete set of SNPs with positive *R*^2^ in the gradient forest (4,198 SNPs with *R*^2^ > 0 out of 6,635 SNPs) and (ii) putatively adaptive SNPs with positive *R*^2^ (1,264 SNPs with *R*^2^ > 0 out of 1,301 SNPs; Table S2). The performance of individual SNP model was assessed by the weighted *R*^2^ value using the *cumimp* function in the R package “gradientForest” [17,37]. The model with the highest *R*^2^ value was considered optimal and used to predict genomic variation under present-day and future climates. For each SNP, the gradient forest was applied with uncorrelated environmental and spatial variables as predictors, with 500 regression trees and a variable correlation threshold of 0.50, as suggested by Fitzpatrick and Keller [17].

The genomic offset measures the mismatch between current and future genomic composition using genotype-environment associations across current gradients as a baseline [17,38]. Populations with a lower genomic offset would be less vulnerable to climate change and require smaller adjustments to track future environmental change. To predict the future genomic composition, we extracted future projections of marine environmental predictors from the Bio-ORACLE v2.0 database [30]. We considered future projections in the 2050s and 2100s under two representative concentration pathway (RCP) scenarios: RCP4.5 (intermediate emission scenario) and RCP8.5 (high emission scenario). Genomic offset was estimated by calculating the Euclidean distance between present-day and future genomic compositions [17]. Furthermore, we conducted a two-tailed Wilcoxon rank-sum test to assess differences in genomic offset between the two clusters.

### 2.3 Species distribution model to predict habitat suitability

In this study, we focused on projecting the habitat suitability of *M. manhattensis* along the Chinese coasts, driven by two primary reasons. Firstly, the invasions of *M. manhattensis* in China occurred in 1976, followed by a rapid spread along the Chinese coasts with dramatically different environmental factors [23,24]. This relatively well-documented invasion history and geographical distribution with varied local environments represent a good system to estimate future invasion risks by integrating genomic offset and habitat suitability at a regional scale. Secondly, niche shifts during biological invasions might occur [32,39,40]. Consequently, invasive species may have different realized niches in invasive compared to their native ranges, making geographical distribution data outside the native ranges valuable for predicting habitat suitability in invaded areas [32]. Indeed, we conducted SDM analyses at the global scale using worldwide distribution data of *M. manhattensis*, and our main conclusions remained consistent (Figs. S9 and S10).

Geographical occurrence records of *M. manhattensis* along Chinese coasts were collected through field sampling, literature sources, and online databases (Table S1). As previous studies suggested [32,41], we removed invalid occurrence points and kept one point per 5-arcmin grid cell (i.e., the spatial resolution of marine predictors). We divided species occurrence records into north and south clusters based on the genetic evidence (see Results for more details). In total, our analyses included 34 records, including 26 and 8 records from the north and south clusters, respectively (Table S1, Fig. S2). The number of species occurrence records can greatly influence the predictive power of SDMs, and the sample size of *M. manhattensis* is sufficient to construct a reliable model [42,43]. We employed the maximum entropy algorithm (MaxEnt) implemented in MaxEnt v3.4.3 to develop SDMs for *M. manhattensis*. MaxEnt has proven to be effective for projecting species habitat suitability, even with small sample sizes [31]. We delineated the study extent by generating a 1,000-km radius buffer around species occurrence records [44] (Fig. S2) and randomly selected 10,000 points within these buffer zones as background data. Previous studies have highlighted the importance of tuning model parameters and avoiding models with default parameterizations [45]. To address this issue, we fitted a total of 32 MaxEnt models with different feature classes and regularization multipliers, and then estimated their predictive performance via a five-fold cross-validation method [46]. We measured MaxEnt's predictive abilities via three metrics: AUC (area under the receiver operating characteristic curve), TSS (true skill statistics), and Boyce (continuous Boyce index). The optimal MaxEnt model was determined based on the validation AUC value and omission rate [47]. To estimate the possible effects of climate change on the invasive potential of *M. manhattensis* in China, we binarized the continuous habitat suitability of SDMs and calculated the change in the suitable range size of *M. manhattensis*. To reduce uncertainties resulting from threshold selection, we quantified range size change via two widely used thresholds: a 10% presence probability threshold and a threshold maximizing the TSS value [48].

### 2.4 Genomic-niche index to combine habitat suitability change and genomic offset

Habitat suitability change and genomic offset evaluate invasion risks of *M. manhattensis* from different aspects. In theory, values of genomic offset are greater than 0, and larger values indicate more vulnerable to future climate change (i.e., lower future invasion risks; [17]). The change in habitat suitability varies from −1 to 1, and positive and negative values suggest increased and decreased invasion risks in the future, respectively. To assess invasion risks comprehensively, we calculated the genomic-niche index by combining habitat suitability change and the genomic offset [19,20]. The genomic-niche index, also termed an eco-genetic index, was first introduced by Chen et al. [20] and has been successfully applied in several works [19,20]. This index can be calculated as follows: $A=B^{\alpha }C^{1-\alpha }$, where *A* represents the genomic-niche index, *B* indicates the change in habitat suitability projected by SDMs, and *C* means genomic offset estimated by gradient forest. The variable *α* is a weight parameter ranging from 0 to 1 [20]. Based on the recommendations by Chen et al. [20], we first scaled habitat suitability change and genomic offset between 0 and 0.9. Subsequently, the optimal *α* value was determined by minimizing the total deviation between the genomic-niche index, change in habitat suitability, and genomic offset. Following the approach outlined by Chen et al. [19], we estimated the optimal *α* value using the artificial bee colony algorithm via the R package “ABCoptim”.

Given that the habitat suitability of *M. manhattensis* along Chinese coasts is expected to decrease in the future (Fig. 3), we focused on regions with decreased habitat suitability. To calculate the genomic-niche index, we used the absolute values of habitat suitability change. This enhances the interpretability of our results, as smaller genomic offset values indicate higher invasion risks, while smaller decreases in habitat suitability (i.e., less reduction in habitat suitability in the future) correspond to higher invasion risks. Thus, the smaller genomic-niche index means a higher overall invasion risk.

## 3 Results

### 3.1 Genomic variation associated with adaptation to local environments

After high-throughput sequencing and data processing, a total of 6,635 high-quality SNPs with a minor allele frequency (MAF) > 0.05 were used in our subsequent analyses. The PCAdapt analysis detected 273 outlier SNPs (*q*-value < 0.05; Fig. 1b). For the LFMM analysis, we initially retained the first four PCs (PC1: 44.03%; PC2: 24.11%; PC3: 17.63%; PC4: 8.29%) based on the environmental PCA. From this analysis, we retained a total of 1,060 significant environment-associated SNPs (*q*-value < 0.05), with 568, 512, 260, and 223 SNPs significantly associated with PC1–4, respectively (Fig. 1b). Collectively, a total of 1,301 SNPs were identified by at least one of the two methods, and they were considered as putatively adaptive SNPs for subsequent analyses.

To characterize the genetic composition of different geographical populations, we used two population assignment analyses: STRUCTURE (Fig. 1c) and PCA (Fig. 1d). Clustering of these putatively adaptive SNPs indicated that populations of *M. manhattensis* along Chinese coasts could be divided into two genetic clusters: the north and south cluster. The north cluster consisted of DAD, LIH, LVS, LAZ, RIZ, and QJZ populations, located in the relatively north of China. The south cluster comprised the ND and XIM populations, situated in the south of China. After the forward selection analysis, two significant variables, i.e., water depth (Dep, *p* = 0.006) and minimum sea surface salinity (Salinity.Min, *p* = 0.029), were finally retained to construct the RDA model. The parsimonious RDA model was significant (*P*-model = 0.031) with an adjusted coefficient of determination (*R*_adj_^2^) of 0.287 (Fig. S3). Altogether, we revealed two putatively adaptive genetic clusters driven by local environments, particularly for water depth and minimum sea surface salinity.

### 3.2 Genomic offset

Among all 6,635 SNPs, we identified 4,198 with positive *R*^2^, significantly associated with environmental and spatial variables. After extracting cumulative importance data, the mean *R*^2^-weighted importance of environmental variables was higher in the putatively adaptive SNPs (0.308) than in the whole SNPs (0.265; Table S2). We thus selected gradient forest models using the dataset of putatively adaptive SNPs to calculate the genomic offset and predict how allele frequencies would shift under future climate change.

**Fig. 2:**
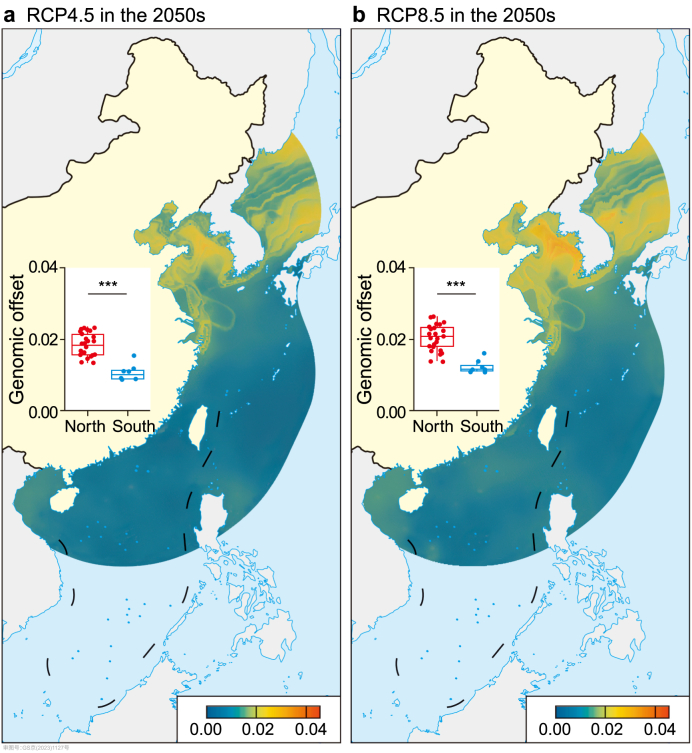
Genomic offset to future climate change under different emission scenarios in the 2050s. **a**, Genomic offset under RCP4.5 in the 2050s. **b**, Genomic offset under RCP8.5 in the 2050s. The comparison of genomic offset between the north and south clusters using the two-tailed Wilcoxon rank-sum test and FDR correction for multiple comparisons. The asterisk (**∗∗∗**) indicates FDR-adjusted *P* < 0.001.

The genomic offset revealed similar spatial patterns under RCP4.5 and RCP8.5 in the 2050s (Fig. 2) and 2100s (Fig. S5). Specifically, the genomic offset decreased from the northern parts (the Bohai Sea and Yellow Sea) to the southern parts (the East and South China Sea) of the distribution regions along Chinese coasts. Our results showed that regardless of future time periods, the genomic offset was significantly greater under the high emission scenario, RCP8.5 (average value: 0.013 ± 0.005 in the 2050s and 0.032 ± 0.011 in the 2100s), than the intermediate emission scenarios, RCP4.5 (average value: 0.012 ± 0.005 in the 2050s and 0.015 ± 0.006 in the 2100s) (Wilcoxon rank sum test, *P* < 0.001; Figs. 2 and S5). Furthermore, a significantly higher level of genomic offset was detected in distributional records belonging to the north than the south clusters (Wilcoxon rank sum test, FDR-adjusted *P* < 0.001; Figs. 2 and S5). Accordingly, populations inhabiting the northern regions of its distribution range (the north cluster) appeared to be more vulnerable to future climate change than populations in the southern part (the south cluster). Altogether, these results indicated that intraspecific variation in genotype-climate associations between the two clusters likely contributed to different degrees of genomic vulnerability to future climate change.

**Fig. 3:**
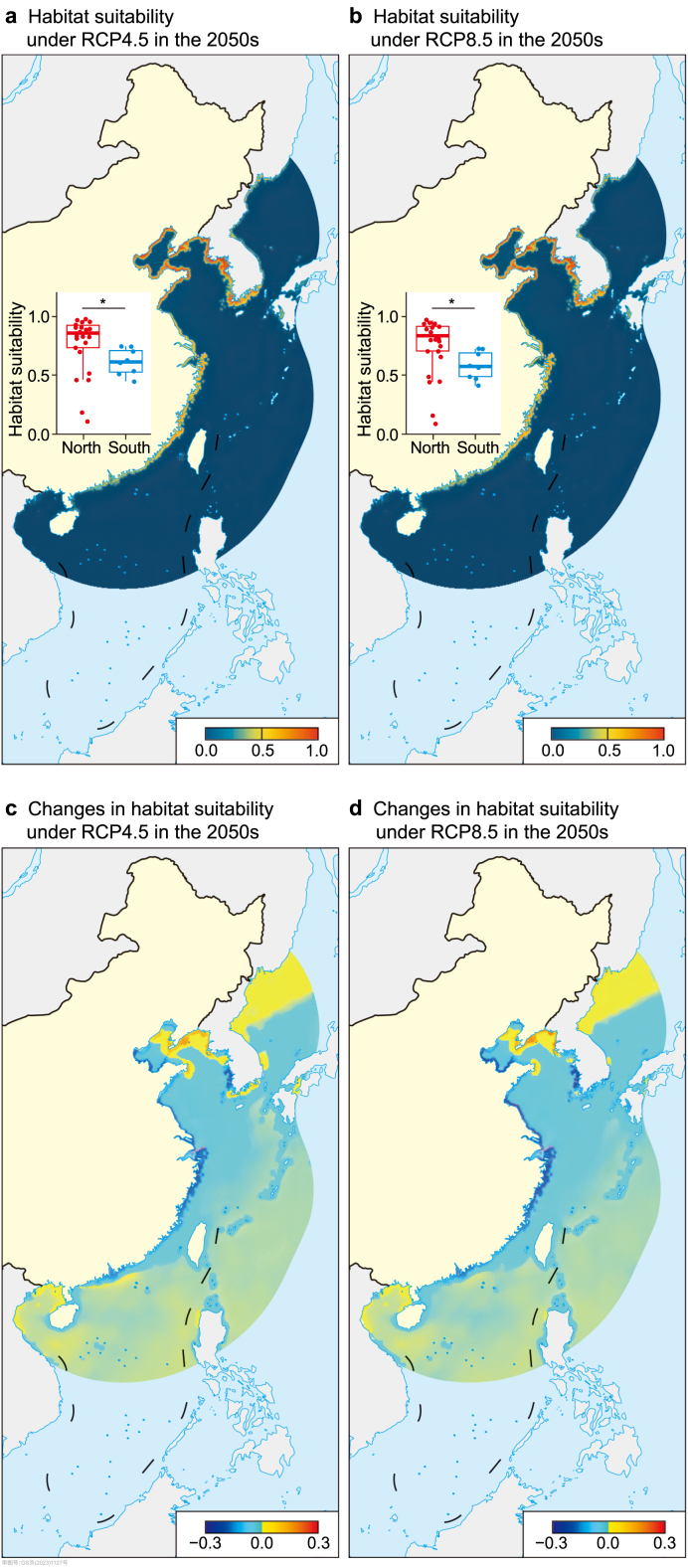
Habitat suitability to future climate change under different emission scenarios in the 2050s. **a**, Habitat suitability under RCP4.5 in the 2050s. **b**, Habitat suitability under RCP8.5 in the 2050s. The comparison of habitat suitability between the north and south clusters using the two-tailed Wilcoxon rank-sum test and FDR correction for multiple comparisons. The asterisk (**∗**) indicates FDR-adjusted *P* < 0.05. **c**, Change in habitat suitability under RCP4.5 in the 2050s. **d**, Change in habitat suitability under RCP8.5 in the 2050s.

### 3.3 Habitat suitability to future climate change

The SDMs for *M. manhattensis* showed high discriminative abilities with AUC, TSS, and Boyce greater than 0.975, 0.926, and 0.328, respectively (Table S3). Under current climatic conditions, most Chinese near-shore shallow coastal waters were predicted to be suitable for this invasive species (Fig. S4). Our modeling results showed that regardless of threshold selection, habitat suitability of *M. manhattensis* along Chinese coasts might reduce remarkably in the future (Figs. 3 and S6), and suitable ranges for this species will reduce, especially in southern China (range contraction varying from −7.385% to −82.514%) (Table S4; Fig. S7). In addition, in the future, the habitat suitability of the north cluster will be significantly higher than that the south cluster (Figs. 3 and S6). These results suggested that the southern regions were likely to experience more habitat suitability reduction than the northern regions in the future.

### 3.4 Genomic-niche index to assess invasion risks

**Fig. 4:**
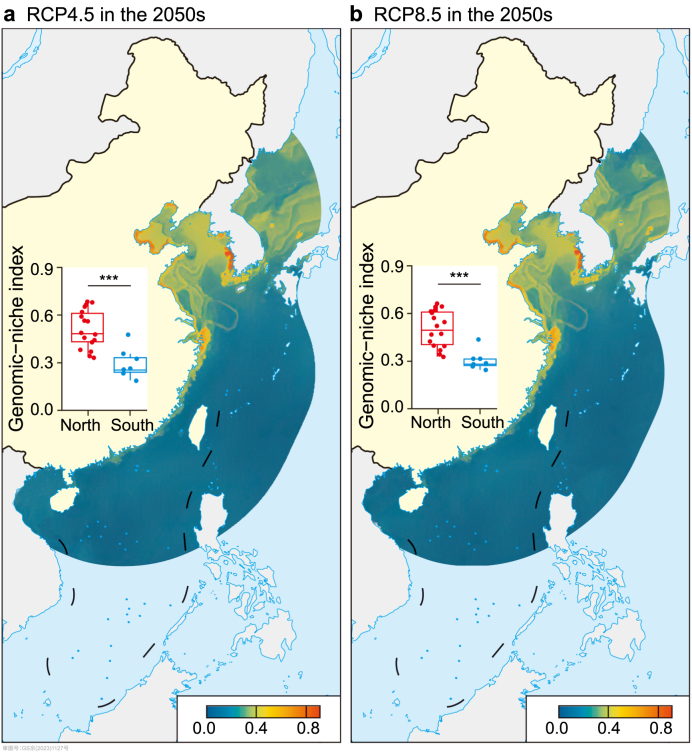
Genomic-niche index based on the combination of genomic offset and change in suitability change to future climate change under different emission scenarios in the 2050s. **a**, Genomic-niche index under RCP4.5 in the 2050s. **b**, Genomic-niche index under RCP8.5 in the 2050s. The comparison of genomic-niche indices between the north and south clusters using the two-tailed Wilcoxon rank-sum test and FDR correction for multiple comparisons. The asterisk (**∗∗∗**) indicates FDR-adjusted *P* < 0.001.

The genomic-niche index exhibited spatial variation along Chinese coasts, and across emission scenarios, the genomic-niche index showed a decreasing trend from north to south along Chinese coasts (Figs. 4 and S8). Most populations inhabiting the northern coasts were forecasted to have medium to high genomic-niche index. In comparison, most southern regions were predicted with a minor genomic-niche index. For example, under RCP4.5 in the 2050s, the genomic-niche index of records belonging to the north cluster ranged from 0.310 to 0.699, with an average of 0.513, while the genomic-niche index of the south cluster ranged from 0.161 to 0.485, with an average of 0.279 (Fig. 4a). Moreover, the south cluster exhibited a significantly lower level of the genomic-niche index than the north cluster (Wilcoxon rank sum test, FDR-adjusted *P* < 0.001; Figs. 4 and S8). These findings indicated that the south cluster would have higher invasion risks in the future.

## 4 Discussion

In this study, we conducted a comprehensive assessment of invasion risks of the invasive *M. manhattensis* by considering adaptive genomic variation and habitat suitability under climate change. The widely used SDMs suggest that populations inhabiting the north should have greater invasion risks than those of the south, while SDMs, together with putatively adaptive genomic variation, provide distinct conclusions. Our findings highlight the importance of incorporating genomic variation in relation to local adaptation into invasion risk assessment. Neglecting genomic information may lead to opposite risk assessment results. Genomic offset and habitat suitability change provide a complementary understanding of invasive potentials under future climate change. Thus, our integrative investigations have valuable implications for effectively managing biological invasions, especially in an era of climate change.

### 4.1 Two clusters driven by local environments

We uncovered two putatively adaptive genetic clusters of *M. manhattensis* along Chinese coasts by using population genomics analyses, such as STRUCTURE (Fig. 1c) and PCA (Fig. 1d). The results are consistent with previous findings using discriminant analysis of principal components (DAPC) [24]. Notably, the boundary of two putatively adaptive clusters in our study does not align with the traditional coastal geographical boundary, i.e., the Yangtze River Estuary Biogeographical Barrier [49]. Mounting evidence in biogeographical and phylogeographical studies has recently indicated novel biogeographical breaks due to the influence of climate change and human activities, e.g., the Subei Biogeographical Barrier [50]. These two putatively adaptive clusters were characterized by distinct environmental conditions, with the north cluster characterized by lower temperature and salinity compared to the south cluster, which exhibited higher temperature and salinity [24]. Based on RDA, we found water depth and minimum sea surface salinity were key drivers in influencing population genetic divergence (Fig. S3). Additionally, we identified several environment-associated genomic loci based on LFMM (Fig. 1b), indicating the signature of environment-driven local adaptation. Recent studies have evidenced that invasive species could rapidly adapt to various environmental conditions in introduced regions [10,23,24,51]. Altogether, the results in this study, together with our previous findings [24], highlight the crucial role of local adaptation to different environments in driving putatively adaptive genomic variation, particularly between the north and south clusters in this study. Interestingly, these two clusters performed differently in the intraspecific response to future climate change.

### 4.2 Incorporating adaptive genomic variation into invasion risk assessment

According to SDM projections, we found that the habitat suitability of the north cluster was significantly higher than the south cluster. Specifically, most regions in the northern parts were predicted to be relatively stable under RCP4.5 and RCP8.5 in the 2050s (Figs. S7a and b). The northern areas are characterized by relatively low temperatures, which may provide a climatic refuge for this temperate-water ascidian in the face of global warming. Conversely, the suitable habitats on the southern coast were predicted to contract under future climate scenarios (Table S4; Figs. 3, S6, and S7). For some southern populations inhabiting warm environments, temperatures may be close to their upper thermal limits in a global warming trend [23]. Our projections were consistent with one recent prediction of range shifts in *M. manhattensis*, and they also forecasted range loss in southern East Asia and a northward range shift in the future [26]. Despite the good predictive performance of our MaxEnt models for *M. manhattensis*, we acknowledge that our MaxEnt models did not consider biotic interactions and physiological information. Future investigations should aim to improve the model by incorporating these factors. Besides, we projected the potential impacts of climate change on the habitat suitability of *M. manhattensis* by future marine climatic layers under RCP scenarios. More information from future studies should be considered under shared socioeconomic pathways.

Researchers have made great efforts to improve the reliability of SDMs in different ways [52,53], making SDMs powerful in macroecological studies. By accounting for genomic information in SDMs, our results show that SDMs can provide a more integrative assessment of invasion risks. Interestingly, we obtained different future invasion risk patterns after combining genomic offset and habitat suitability change. For the south cluster, the effect of suitable habitat loss under future climates might be mitigated by the minor genomic offset (Fig. 2, Fig. 4). Rapid genetic adaptation to different environments predominantly depends on standing genetic variation, and those preadapted individuals may be genetically equipped to withstand and adapt to future climate change [16,54]. One previous study in a marine tunicate invader, *Ciona robusta*, has indicated that rapid microevolution played a key role in adapting to high temperature and salinity environments during recent range expansions to the Red Sea [25]. We infer that the south cluster may possess preadapted genotypes, which would contribute to overcoming the challenge of ecological constraints under future climate change [24]. As a result, we expect a northward shift of the south cluster along Chinese coasts in the future. While the genomic offset approach provides valuable insights into the magnitude of climate change disruption, it also makes some simplified assumptions that should be used cautiously in applied studies [55]. We acknowledge that the genetic offset of this ascidian may be overestimated, and the potential of future invasion risk may be underestimated due to the assumption that the genetic makeup of the population in future climates remains unchanged accordingly. Future studies are still needed to include estimating genomic offset and further performing experimental testing and functional validation of fitness (such as common garden experiments) [55].

Our findings indicate that habitat suitability change and genomic offset may functionally complement predicting future invasion risks. This finding is consistent with a recent study on a globally invasive pest diamondback moth *Plutella xylostella* [20]. They found that those populations in central Africa and southern China were predicted with reduced habitat suitability and minor genetic offset under future climates [20]. SDMs and gradient forest models may provide different predictive results due to their distinct perspectives [56]. Altogether, our results highlight that prediction results merely obtained from a single approach may be incomplete, and the comprehensive index in our study, by combining adaptive genomic variation and SDMs, would provide more comprehensive insights into potential responses and invasion risks of specific populations to climate change.

Performing invasion risk assessment under future climate change contributes to identifying invasion hotspots and guides the management of invasive species [57]. Consistent with previous studies [13,14], our results also support the conclusion that different geographical populations or genetic clusters may respond differently to future climate change. Future work should integrate distinct analyses to synthetically evaluate invasion risks, not only by combining habitat suitability and adaptive genomic variation but also by incorporating additional aspects of adaptive capacity into predictive models. These factors may include genomic structural variation, gene expression plasticity, and epigenetic changes, which have been shown to be responsible for range expansions during invasions [[58], [59], [60]].

## CRediT author contribution statement

**Yiyong Chen:** Conceptualization, Methodology, Software, Validation, Formal Analysis, Investigation, Writing - Original Draft, Writing - Review & Editing, Visualization. **Yangchun Gao:** Methodology, Software, Formal Analysis, Investigation. **Xuena Huang:** Validation, Investigation. **Shiguo Li:** Methodology, Investigation. **Zhixin Zhang:** Methodology, Software, Validation, Formal Analysis, Investigation, Writing - Original Draft, Writing - Review & Editing, Visualization. **Aibin Zhan:** Conceptualization, Writing - Review & Editing, Supervision, Project Administration, Funding Acquisition.

## Declaration of competing interest

The authors declare that they have no known competing financial interests or personal relationships that could have appeared to influence the work reported in this paper.
