# Supplementary material for: Incorporating adaptive genomic variation into predictive models for invasion risk assessment
Source: Environ Sci Ecotechnol. 2023 Jul 11;18:100299. doi: 10.1016/j.ese.2023.100299 (PMC10494315; doi:10.1016/j.ese.2023.100299)

**Appendix S1**

**Table S1** Occurrence records of *Molgula manhattensis* along Chinese coasts available from field sampling, published literature, and public database.

| **Location** | **Province** | **longitude** | **latitude** | **Year** | **Cluster** | **Reference** |
| --- | --- | --- | --- | --- | --- | --- |
| Jinzhou | Liaoning | 121.16 | 40.89 | 2013 | north | Field sampling; Chen Y, Li S, Lin Y, et al. Population genetic patterns of the solitary tunicate, *Molgula manhattensis*, in invaded Chinese coasts: large-scale homogeneity but fine-scale heterogeneity. Marine Biodiversity, 2018, 48(4): 2137-2149. |
| Linghai | Liaoning | 121.33 | 40.88 | 2013 | north | Field sampling; Chen Y, Li S, Lin Y, et al. Population genetic patterns of the solitary tunicate, *Molgula manhattensis*, in invaded Chinese coasts: large-scale homogeneity but fine-scale heterogeneity. Marine Biodiversity, 2018, 48(4): 2137-2149. |
| Dandong | Liaoning | 124.30 | 39.87 | 2013 | north | Field sampling; Chen Y, Li S, Lin Y, et al. Population genetic patterns of the solitary tunicate, *Molgula manhattensis*, in invaded Chinese coasts: large-scale homogeneity but fine-scale heterogeneity. Marine Biodiversity, 2018, 48(4): 2137-2149. |
| Zhuanghe | Liaoning | 122.99 | 39.66 | 2013 | north | Field sampling; Chen Y, Li S, Lin Y, et al. Population genetic patterns of the solitary tunicate, *Molgula manhattensis*, in invaded Chinese coasts: large-scale homogeneity but fine-scale heterogeneity. Marine Biodiversity, 2018, 48(4): 2137-2149. |
| Laoting | Hebei | 119.10 | 39.27 | 2013 | north | Field sampling; Chen Y, Li S, Lin Y, et al. Population genetic patterns of the solitary tunicate, *Molgula manhattensis*, in invaded Chinese coasts: large-scale homogeneity but fine-scale heterogeneity. Marine Biodiversity, 2018, 48(4): 2137-2149. |
| Changhaixian | Liaoning | 122.59 | 39.26 | 2013 | north | Field sampling; Chen Y, Li S, Lin Y, et al. Population genetic patterns of the solitary tunicate, *Molgula manhattensis*, in invaded Chinese coasts: large-scale homogeneity but fine-scale heterogeneity. Marine Biodiversity, 2018, 48(4): 2137-2149. |
| Dengshahe | Liaoning | 122.11 | 39.21 | 2013 | north | Field sampling; Chen Y, Li S, Lin Y, et al. Population genetic patterns of the solitary tunicate, *Molgula manhattensis*, in invaded Chinese coasts: large-scale homogeneity but fine-scale heterogeneity. Marine Biodiversity, 2018, 48(4): 2137-2149. |
| Hebei | Hebei | 119.06 | 39.16 | 2013 | north | Field sampling; Chen Y, Li S, Lin Y, et al. Population genetic patterns of the solitary tunicate, *Molgula manhattensis*, in invaded Chinese coasts: large-scale homogeneity but fine-scale heterogeneity. Marine Biodiversity, 2018, 48(4): 2137-2149. |
| Tianjin | Tianjin | 117.80 | 39.08 | 2013 | north | Literature survey; Han S, Cao W, Chen D, et al. Review of fouling ascidians in the coastal waters of China. Ecological Science, 2018, 37(1): 186-191. |
| Bohai Gulf | Tianjin | 117.84 | 39.05 | NA | north | Literature survey; Yan T, Cao W. Ecology of marine fouling organism in Huanghai and Bohai Seas. Journal of Marine Sciences, 2008, 3: 015. |
| Dalian | Liaoning | 121.78 | 39.02 | NA | north | Literature survey; Cao S, Zhang C, Zuo X. The effect of different ecological factors on oxygen consumption in ascidian *Molgula manhattensis*. Journal of Dalian Ocean University, 2012, 3: 006. |
| Tanggu | Tianjin | 117.82 | 38.98 | 1976 | north | Literature survey; Huang Z G, Zheng C X, Lin S, et al. Fouling organisms at Daya Bay nuclear power station, China. The Marine Biology of the South China Sea. Hong Kong University Press, Hong Kong, 1993: 121-130. |
| Heishujiao | Liaoning | 121.56 | 38.88 | 2013 | north | Field sampling; Chen Y, Li S, Lin Y, et al. Population genetic patterns of the solitary tunicate, *Molgula manhattensis*, in invaded Chinese coasts: large-scale homogeneity but fine-scale heterogeneity. Marine Biodiversity, 2018, 48(4): 2137-2149. |
| Liaodong | Liaoning | 121.69 | 38.87 | NA | north | Literature survey; Yan T, Cao W. Ecology of marine fouling organism in Huanghai and Bohai Seas. Journal of Marine Sciences, 2008, 3: 015. |
| Lvshun | Liaoning | 121.15 | 38.47 | 2013 | north | Field sampling; Chen Y, Li S, Lin Y, et al. Population genetic patterns of the solitary tunicate, *Molgula manhattensis*, in invaded Chinese coasts: large-scale homogeneity but fine-scale heterogeneity. Marine Biodiversity, 2018, 48(4): 2137-2149. |
| Laizhou | Shandong | 120.32 | 37.86 | 2013 | north | Field sampling; Chen Y, Li S, Lin Y, et al. Population genetic patterns of the solitary tunicate, *Molgula manhattensis*, in invaded Chinese coasts: large-scale homogeneity but fine-scale heterogeneity. Marine Biodiversity, 2018, 48(4): 2137-2149. |
| Penglai | Shandong | 120.75 | 37.81 | 2001 | north | [Literature survey; https://invasions.si.edu/nemesis/species_summary/159557](https://invasions.si.edu/nemesis/species_summary/159557) |
| Zhifu Bay | Shandong | 121.38 | 37.57 | 2001 | north | [Literature survey; https://invasions.si.edu/nemesis/species_summary/159557](https://invasions.si.edu/nemesis/species_summary/159557) |
| Shandong | Shandong | 122.15 | 37.56 | NA | north | Literature survey; Yan T, Cao W. Ecology of marine fouling organism in Huanghai and Bohai Seas. Journal of Marine Sciences, 2008, 3: 015. |
| Yantai | Shandong | 121.45 | 37.52 | 2013 | north | Field sampling; Chen Y, Li S, Lin Y, et al. Population genetic patterns of the solitary tunicate, *Molgula manhattensis*, in invaded Chinese coasts: large-scale homogeneity but fine-scale heterogeneity. Marine Biodiversity, 2018, 48(4): 2137-2149. |
| Qingdao | Shandong | 120.34 | 36.08 | 2001 | north | [Literature survey; https://invasions.si.edu/nemesis/species_summary/159557](https://invasions.si.edu/nemesis/species_summary/159557) |
| Rizhao | Shandong | 119.69 | 35.57 | 2013 | north | Field sampling; Chen Y, Li S, Lin Y, et al. Population genetic patterns of the solitary tunicate, *Molgula manhattensis*, in invaded Chinese coasts: large-scale homogeneity but fine-scale heterogeneity. Marine Biodiversity, 2018, 48(4): 2137-2149. |
| Lianyungang | Jiangsu | 119.38 | 34.78 | 2013 | north | Literature survey; Han S, Cao W, Chen D, et al. Review of fouling ascidians in the coastal waters of China. Ecological Science, 2018, 37(1): 186-191. |
| Liangyungang | Jiangsu | 119.29 | 34.71 | 2001 | north | [Literature survey; https://invasions.si.edu/nemesis/species_summary/159557](https://invasions.si.edu/nemesis/species_summary/159557) |
| Qingjiangzhen | Zhejiang | 121.17 | 28.29 | 2013 | north | Field sampling; Chen Y, Li S, Lin Y, et al. Population genetic patterns of the solitary tunicate, *Molgula manhattensis*, in invaded Chinese coasts: large-scale homogeneity but fine-scale heterogeneity. Marine Biodiversity, 2018, 48(4): 2137-2149. |
| Zhejiang | Zhejiang | 121.07 | 28.16 | 2013 | north | Field sampling; Chen Y, Li S, Lin Y, et al. Population genetic patterns of the solitary tunicate, *Molgula manhattensis*, in invaded Chinese coasts: large-scale homogeneity but fine-scale heterogeneity. Marine Biodiversity, 2018, 48(4): 2137-2149. |
| Ningde | Fujian | 119.62 | 26.63 | 2008 | south | Literature survey; Fan C, Lu Y, Zhai S, et al. On fatty acid composition of three major ascidians from East China Sea. Marine Fisheries, 2010,32:109-112 |
| Fujian | Fujian | 119.63 | 26.38 | 2013 | south | Field sampling; Chen Y, Li S, Lin Y, et al. Population genetic patterns of the solitary tunicate, *Molgula manhattensis*, in invaded Chinese coasts: large-scale homogeneity but fine-scale heterogeneity. Marine Biodiversity, 2018, 48(4): 2137-2149. |
| Luoyuan Bay | Fujian | 119.54 | 26.04 | 2001 | south | [Literature survey; https://invasions.si.edu/nemesis/species_summary/159557](https://invasions.si.edu/nemesis/species_summary/159557) |
| Xiamen | Fujian | 118.49 | 24.53 | NA | south | Literature survey; Zheng C X. Ascidians of fouling organisms in the Yellow Sea and Bohai Sea. Acta Zool Sin, 1988, 34(2): 180-187. |
| Xiamen Harbor | Fujian | 118.08 | 24.46 | 2001 | south | [Literature survey; https://invasions.si.edu/nemesis/species_summary/159557](https://invasions.si.edu/nemesis/species_summary/159557) |
| Dongshan Bay | Fujian | 117.43 | 23.70 | 2001 | south | [Literature survey; https://invasions.si.edu/nemesis/species_summary/159557](https://invasions.si.edu/nemesis/species_summary/159557) |
| Shantou Harbor | Guangdong | 116.67 | 23.37 | 2001 | south | [Literature survey; https://invasions.si.edu/nemesis/species_summary/159557](https://invasions.si.edu/nemesis/species_summary/159557) |
| Daya Bay | Guangdong | 114.65 | 22.70 | NA | south | Literature survey; Huang Z G, Zheng C X, Lin S, et al. Fouling organisms at Daya Bay nuclear power station, China. The Marine Biology of the South China Sea. Hong Kong University Press, Hong Kong, 1993: 121-130. |

**Table S2** Summary of two single nucleotide polymorphism (SNP) datasets used to fit gradient forest models and associated metrics of model performance. All R^2^ positive SNPs were based on the complete set of SNPs (4,198 SNPs with R^2^ >0 from all 6,635 SNPs). Putatively adaptive SNPs were based on 1,264 SNPs with R^2^ >0 from 1,301 putatively adaptive SNPs.

| **SNPs** | **Mean R^2^** | **Range** |
| --- | --- | --- |
| All R^2^ positive SNPs | 0.265 | 0.00008-0.64561 |
| Putatively adaptive SNPs | 0.308 | 0.00006-0.66900 |

**Table S3** Predictive performance (average ± standard deviation) of species distribution models for *Molgula manhattensis*.

| **Feature class** | **Regularization multiplier** | **AUC** | **TSS** | **Boyce** | **10% presence probability** | **Threshold maximizing TSS value** |
| --- | --- | --- | --- | --- | --- | --- |
| Hinge | 3.5 | 0.975 ± 0.010 | 0.926 ± 0.054 | 0.328 ± 0.318 | 0.580 | 0.310 |

**Table S4** Range size change (%) of *Molgula manhattensis* in the future projected by species distribution models.

| **RCP** | **Future period** | **10% presence probability** | **Threshold maximizing TSS** |
| --- | --- | --- | --- |
| RCP4.5 | 2050 | -23.021 | -7.385 |
| RCP8.5 | 2050 | -29.229 | -10.835 |
| RCP4.5 | 2100 | -34.609 | -12.383 |
| RCP8.5 | 2100 | -82.514 | -31.635 |

**Table S5** List of abbreviations used in this study.

| **Abbreviations** | **Definitions** |
| --- | --- |
| SDMs | Species distribution models |
| SNPs | Single nucleotide polymorphisms |
| LFMM | Latent factor mixed-effect model |
| DAPC | Discriminant analysis of principal components |
| FDR | False discovery rate |
| sNMF | Sparse non-negative matrix factorization |
| RDA | Redundancy analysis |
| PCNMs | Principal coordinates of neighborhood matrices |
| RCP | Representative concentration pathway |
| MaxEnt | Maximum entropy algorithm |
| AUC | Area under the receiver operating characteristic curve |
| TSS | True skill statistics |
| MAF | Minor allele frequency |
| ABC | Artificial bee colony |

**Figure S1** The pairwise Pearson’s correlation coefficients (*r*) among 14 marine predictors. dshore: distance to shore; depth: water depth; SST: sea surface temperature; SSS: sea surface salinity; Lt: long-term; max: maximum value; min: minimum value. Predictors are highly correlated when |*r*| > 0.70.


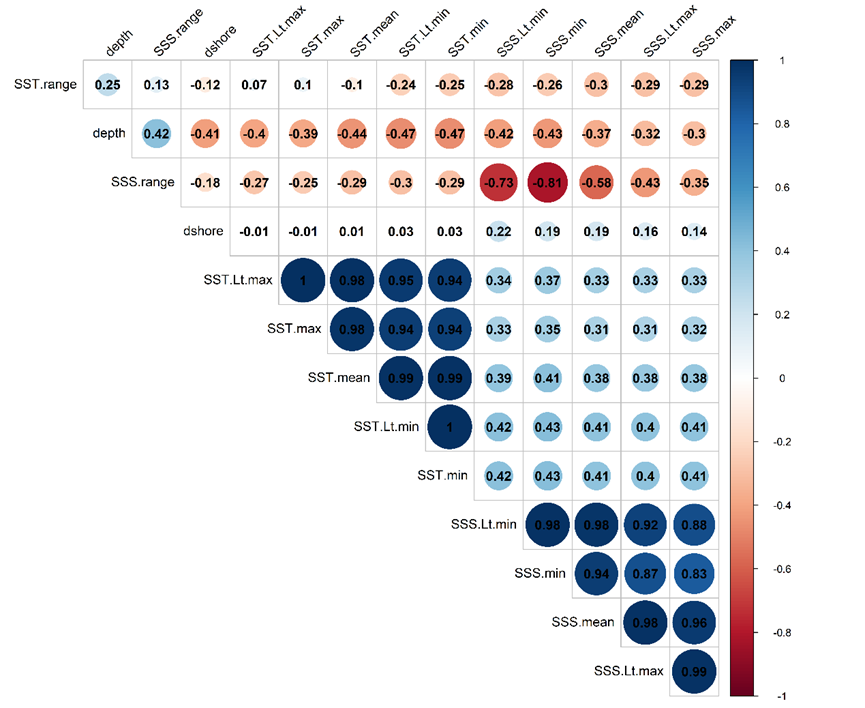


**Figure S2** Occurrence records of *Molgula manhattensis* along Chinese coasts used for species distribution model development. The red and blue points represent occurrence records for the north and south clusters, respectively. The black dashed lines correspond to 1,000 km radius buffer around occurrence records.


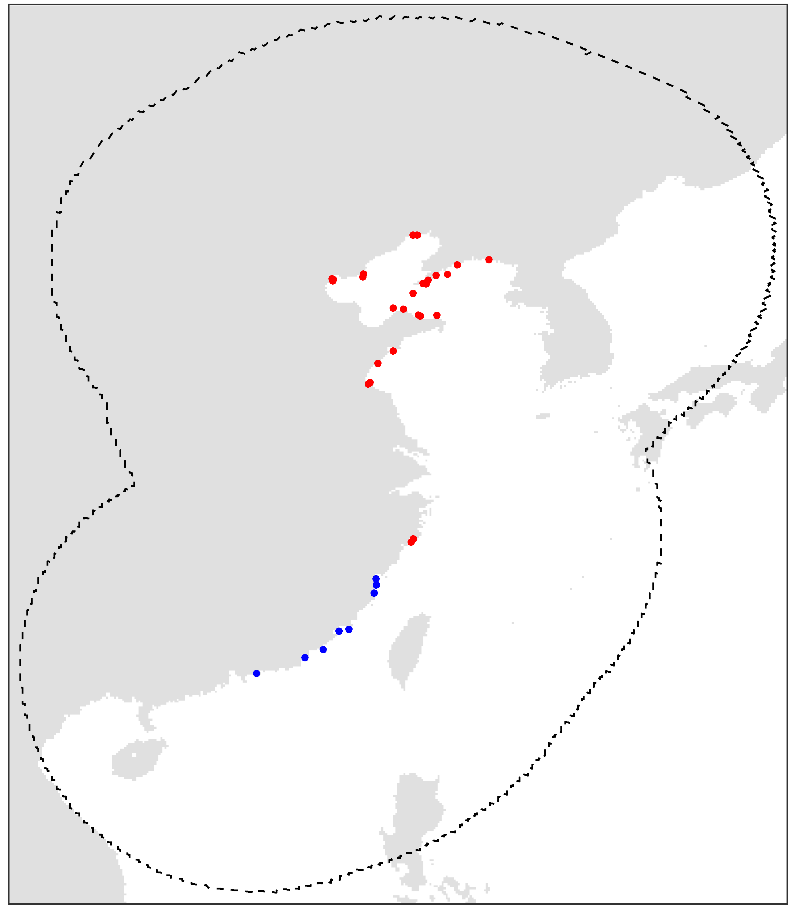


**Figure S3** Redundancy analysis plot based on putatively adaptive single nucleotide polymorphisms (SNPs)**.** Dep: water depth; Salinity.Min: minimum sea surface salinity; Adj.R2: adjusted coefficient of determination; P: the significance of the RDA model.


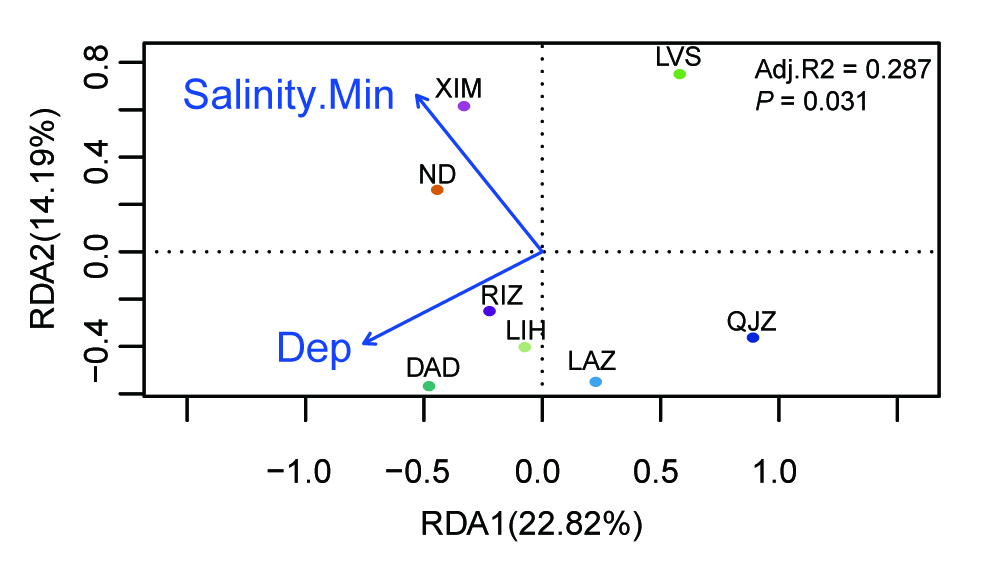


**Figure S4** Habitat suitability of *Molgula manhattensis* under present-day conditions. (a) Continuous habitat suitability prediction. (b) Binary habitat suitability using 10% presence probability threshold. (c) Binary habitat suitability using threshold maximizing TSS value.


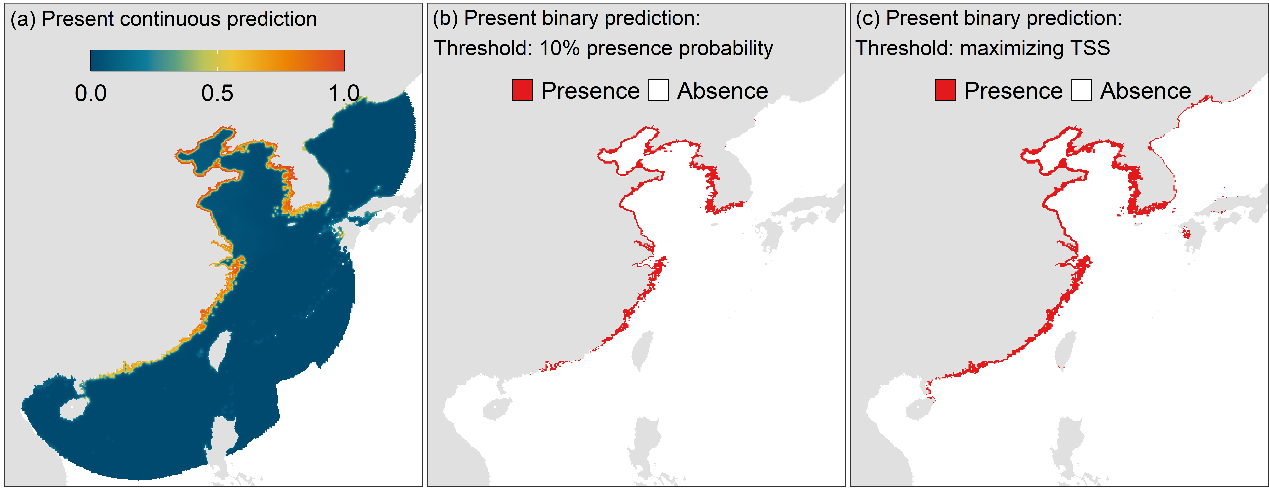


**Figure S5** Genomic offset to future climate change under different emission scenarios in the 2100s. **a.** Genomic offset under RCP4.5 in the 2100s. **b.** Genomic offset under RCP8.5 in the 2100s. The comparison of genomic offset between the north and souths using the two-tailed Wilcoxon rank-sum test and FDR correction for multiple comparisons. The asterisk (***) indicates FDR-adjusted *P* < 0.001.


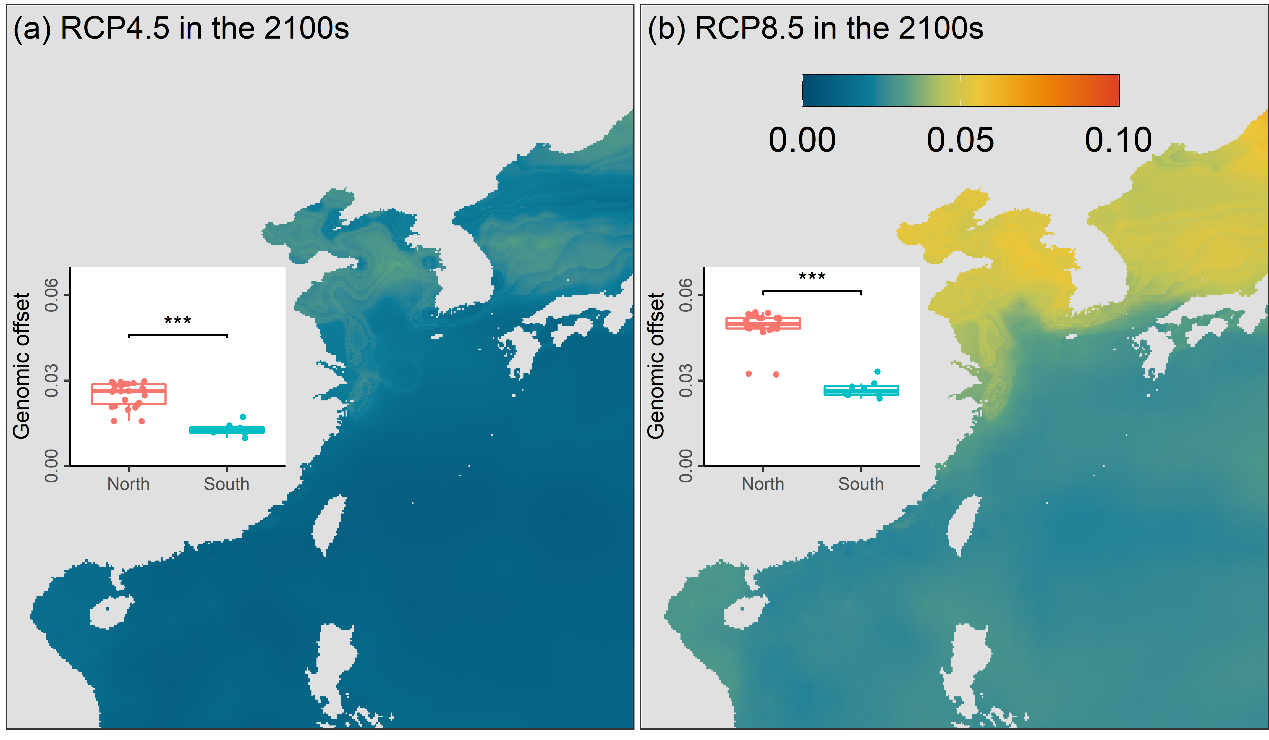


**Figure S6** Habitat suitability to future climate change under different emission scenarios in the 2100s. **a.** Habitat suitability under RCP4.5 in the 2100s. **b.** Habitat suitability under RCP8.5 in the 2100s. The comparison of habitat suitability between the north and souths using the two-tailed Wilcoxon rank-sum test and FDR correction for multiple comparisons. The asterisk (*****) indicates FDR-adjusted *P* < 0.05. **c.** Change in habitat suitability under RCP4.5 in the 2100s. **d.** Change in habitat suitability under RCP8.5 in the 2100s.


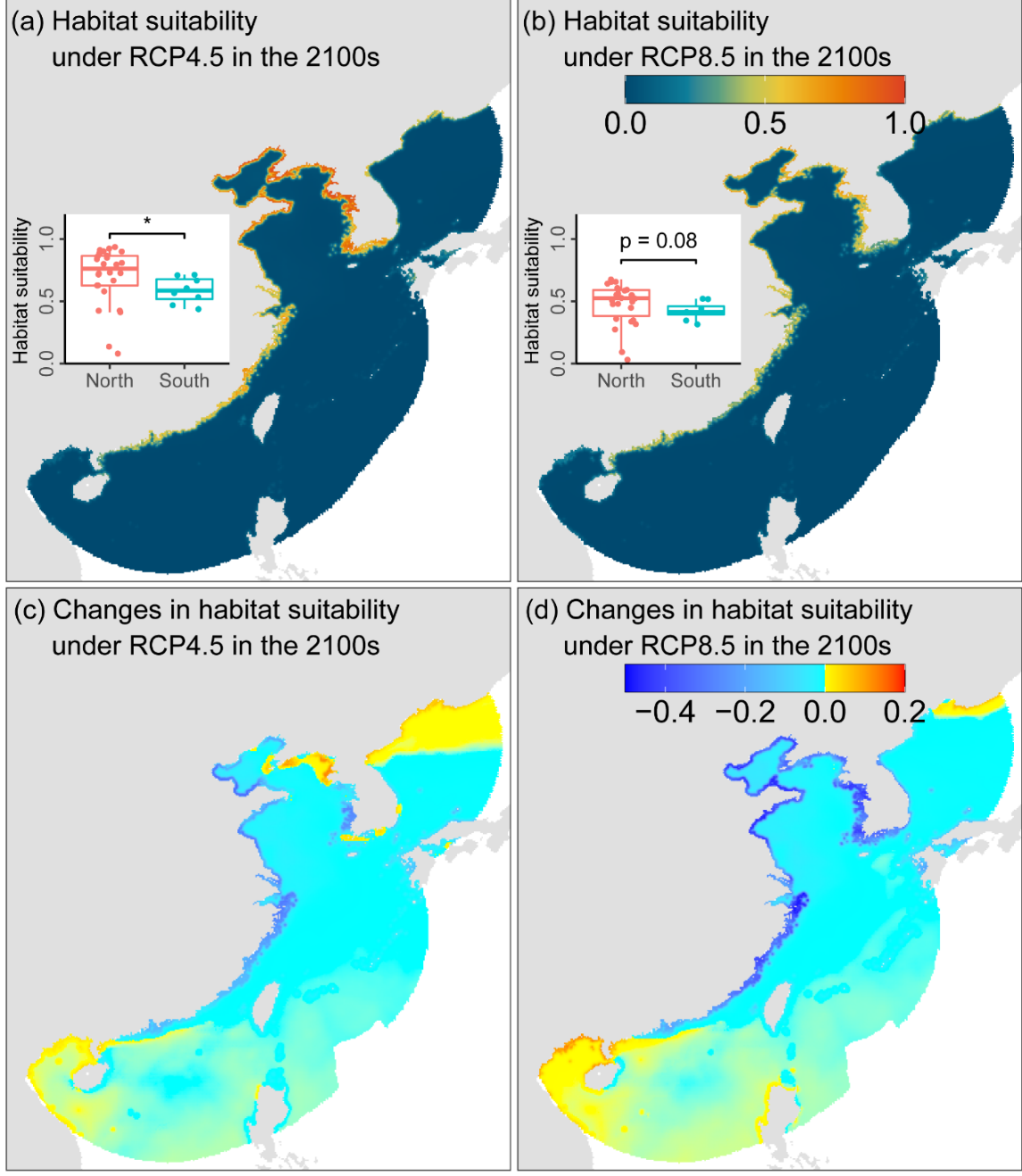


**Figure S7** Change in binary habitat suitability under different emission scenarios in the 2050s and 2100s. Loss: areas predicted to be suitable under present-day conditions but unsuitable in the future; Stable: areas predicted to be suitable under both present-day and future climatic conditions; Gain: areas predicted to be unsuitable under present-day conditions but suitable in the future.


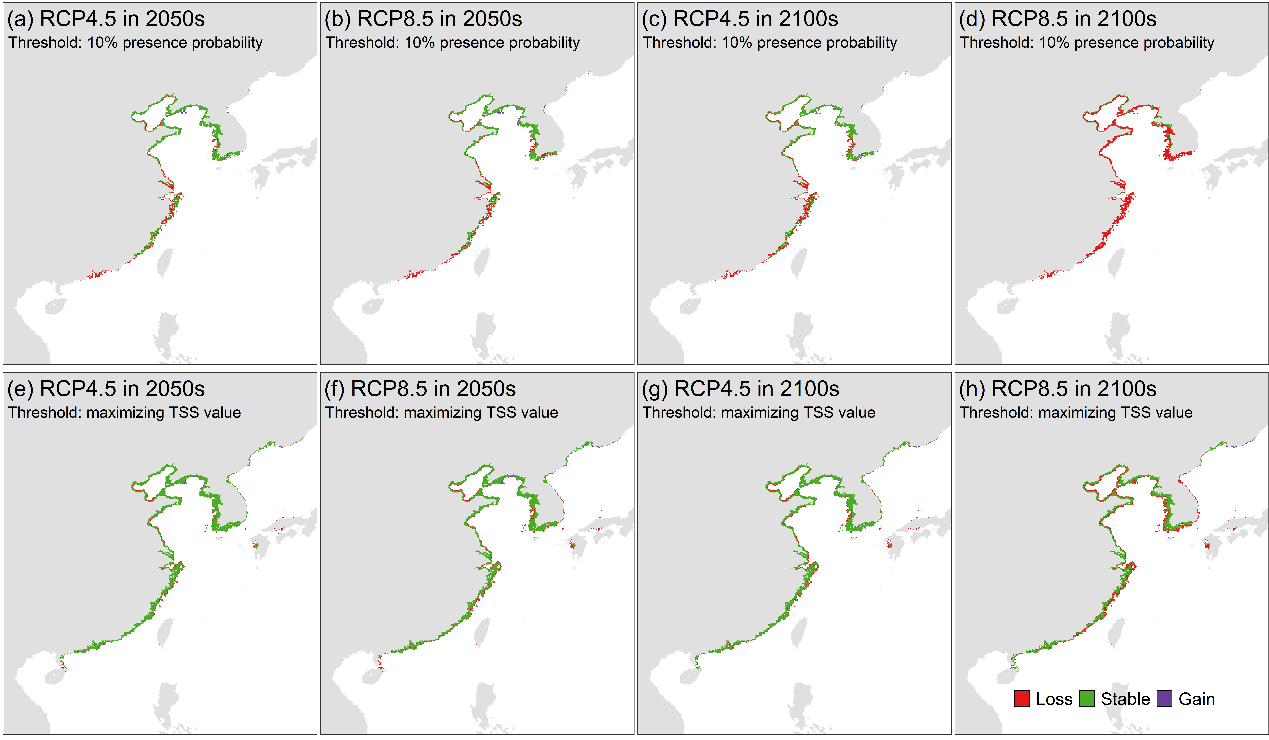


**Figure S8** Genomic-niche index based on the combination of genomic offset and change in suitability change to future climate change under different emission scenarios in the 2100s. **a.** Genomic-niche index under RCP4.5 in the 2100s. **b.** Genomic-niche index under RCP8.5 in the 2100s. The comparison of genomic-niche index between the north and souths using the two-tailed Wilcoxon rank-sum test and FDR correction for multiple comparisons. The asterisk (*******) indicates FDR-adjusted *P* < 0.001.


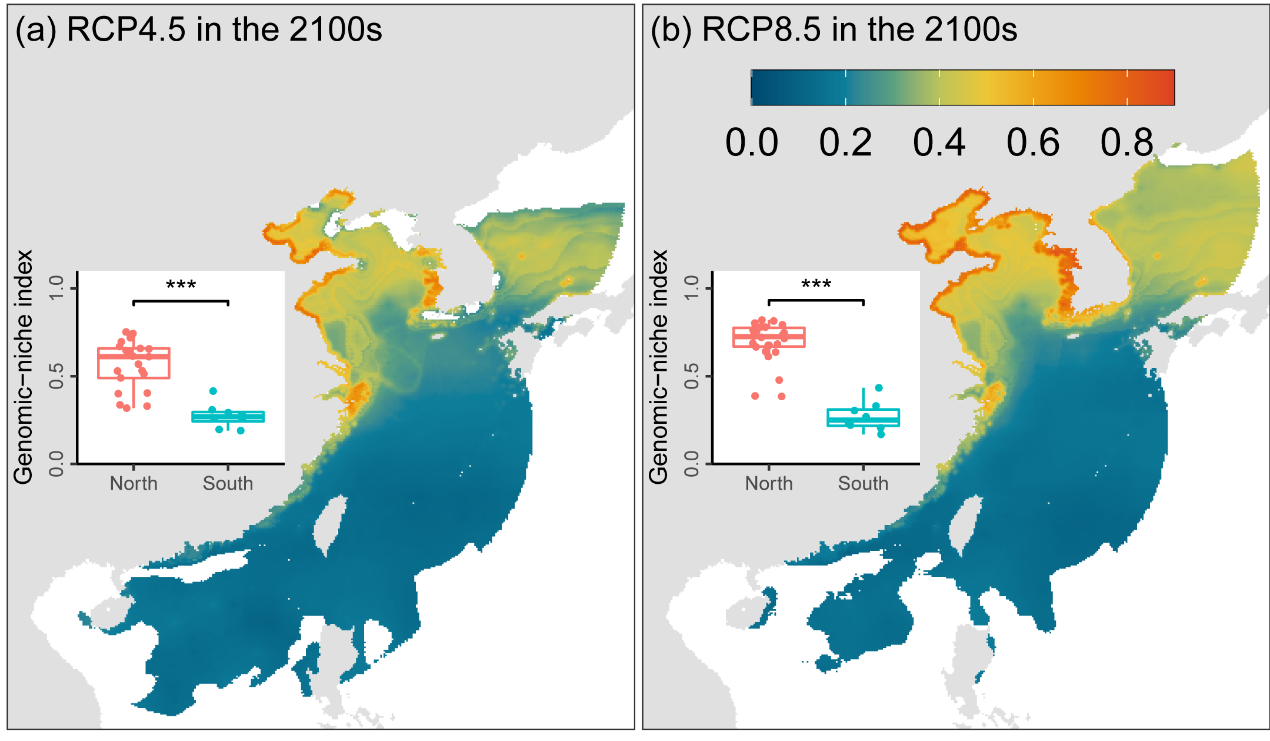


**Figure S9** Habitat suitability and genomic-niche index projections under different emission scenarios in the 2050s by using the global distribution dataset. The global occurrence records of *Molgula manhattensis* was from Zhang et al. (2020), and the conclusions based on global dataset were identical to those based on Chinese dataset.


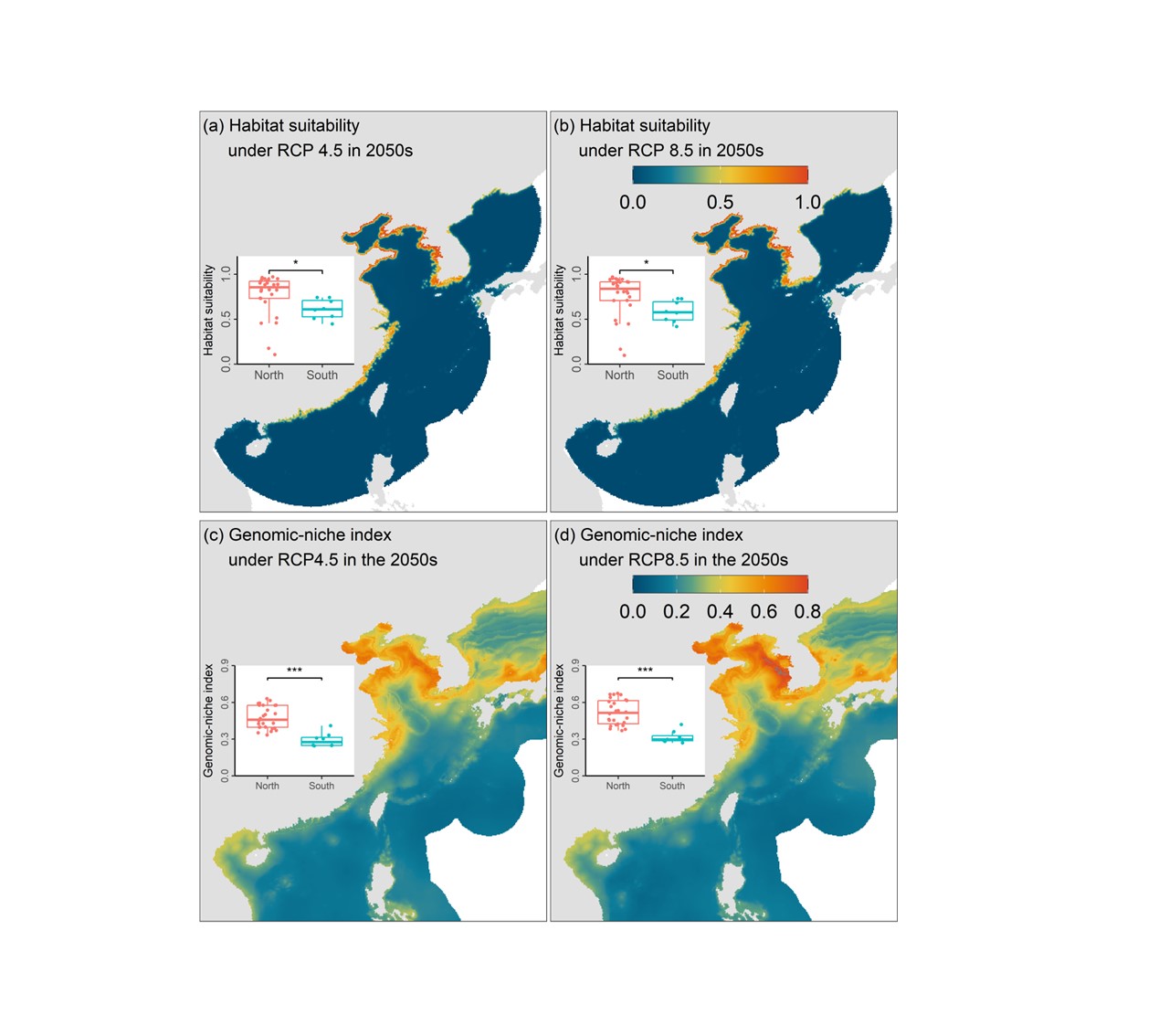


**Figure S10** Habitat suitability and genomic-niche index projections under different emission scenarios in the 2100s by using the global distribution dataset. The global occurrence records of *Molgula manhattensis* was from Zhang et al. (2020), and the conclusions based on global dataset were identical to those based on Chinese dataset.


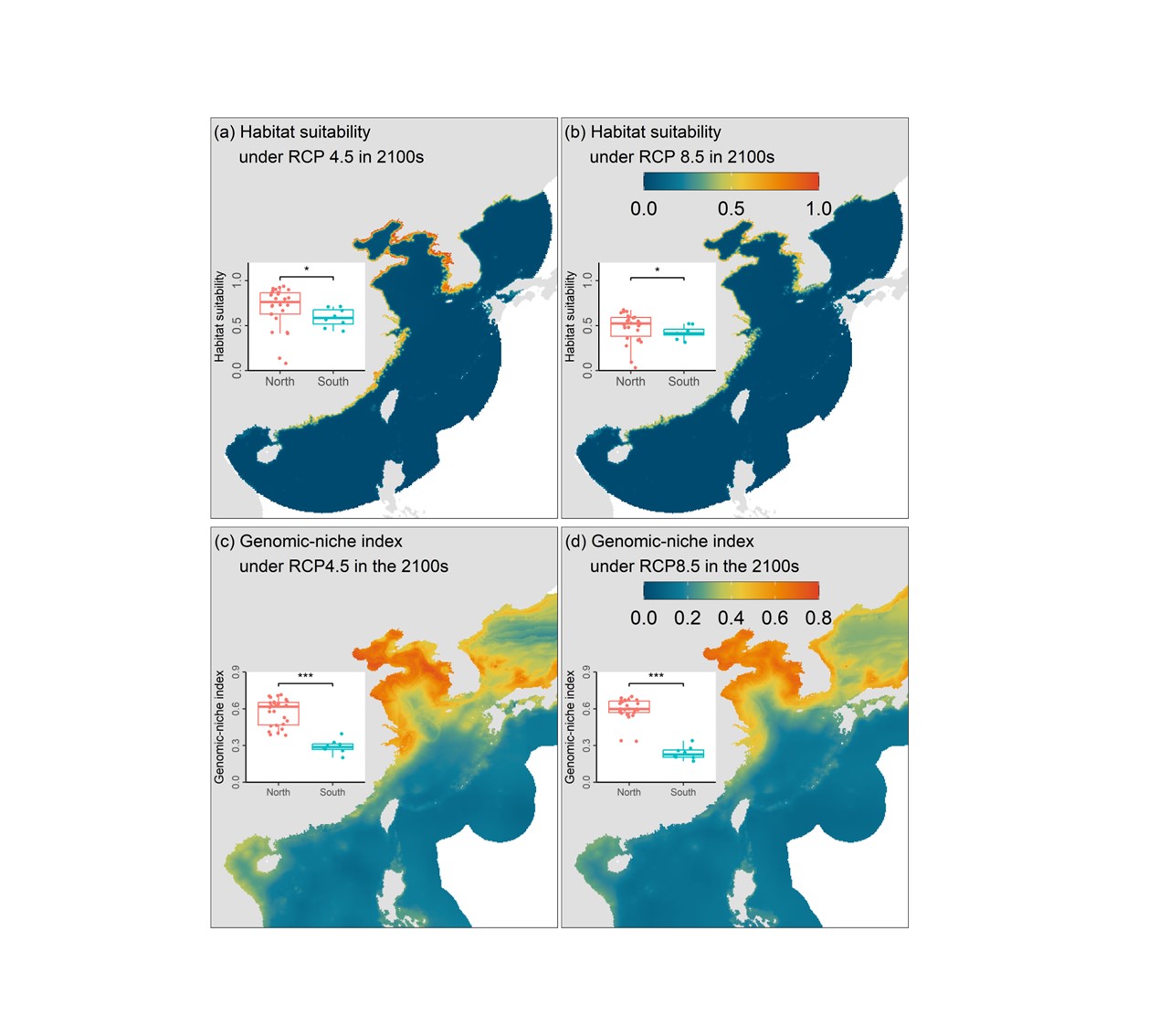

Supplement: Multimedia component 1 [file mmc1.docx]
